# Human embryonic mesenchymal lung-conditioned medium promotes differentiation to myofibroblast and loss of stemness phenotype in lung adenocarcinoma cell lines

**DOI:** 10.1186/s13046-021-02206-z

**Published:** 2022-01-26

**Authors:** Jordi Canals, Alfons Navarro, Cristina Vila, Josep M. Canals, Tania Díaz, Melissa Acosta-Plasencia, Coralí Cros-Font, Bing Han, Yangyi He, Mariano Monzó

**Affiliations:** 1 Molecular Oncology and Embryology Laboratory, Human Anatomy Unit, Faculty of Medicine and Health Sciences, University of Barcelona, IDIBAPS, Barcelona, Spain; 2 Thoracic Oncology Unit, Hospital Clinic, Barcelona, Spain; 3 Laboratory of Stem Cells and Regenerative Medicine, Department of Biomedical Sciences, Creatio, Production and Validation Center of Advanced Therapies, Faculty of Medicine and Health Sciences, Institute of Neurosciences, University of Barcelona, Barcelona, Spain; 4 August Pi i Sunyer Biomedical Research Institute (IDIBAPS), Barcelona, Spain; 5 Networked Biomedical Research Centre for Neurodegenerative Disorders (CIBERNED), Barcelona, Spain; 6 University of Cheng Du, Cheng Du, 610106 China

**Keywords:** NSCLC, Lung development, Embryonic microenvironment, Reprogramming, Myofibroblast, OSKM, NKX2-1, MYH

## Abstract

**Background:**

When genes responsible for normal embryonic development are abnormally expressed in adults, it can lead to tumor development. This can suggest that the same mechanism that controls embryonic differentiation can also control tumor differentiation. We hypothesize that the malignant phenotype of lung cancer cells could acquire benign characteristics when in contact with an embryonic lung microenvironment. We cultured two lung cancer cell lines in embryonic lung mesenchyme-conditioned medium and evaluated morphological, functional and molecular changes.

**Methods:**

The human embryonic mesenchymal lung-conditioned medium (hEML-CM) was obtained by culturing lung cells from embryos in the pseudoglandular stage of development. The NSCLC cell lines A549 and H1299 we cultured in the hEML-CM and in a tumor-conditioned medium. Morphological changes were analyzed with optical and transmission electron microscopy. To evaluate the functional effect of conditioned medium in tumor cells, we analyzed cell proliferation, migration, colony formation capacity in 2D and 3D and in vivo tumor growth capacity. The expression of the pluripotency genes OSKM, the adenocarcinoma marker NKX2-1, the lung surfactant proteins SFTP, the myofibroblast marker MYH and DNMT3A/3B was analyzed with qRT-PCR and the presence of the myofibroblast markers vimentin and α-SMA with immunofluorescence. Transcriptomic analysis was performed using Affymetrix arrays.

**Results:**

The A549 and H1299 cells cultured in hEML-CM lost their epithelial morphology, acquired mesodermal characteristics, and decreased proliferation, migration, and colony formation capacity in 2D and 3D, as well as reduced its capacity to growth in vivo. The expression of OSKM, NKX2-1 and SFTP decreased, while that of DNMT3A/3B, vimentin, α-SMA and MYH increased. Distant matrix analysis based on transcriptomic profile showed that conditioned cells were closer to myoblast and human lung fibroblast than to normal epithelial immortalized lung cells. A total of 1631 for A549 and 866 for H1299 differentially expressed genes between control and conditioned cells were identified.

**Conclusions:**

To the best of our knowledge, this is the first study to report that stimuli from the embryonic lung can modulate the malignant phenotype of lung cancer cells, control their growth capacity and activate their differentiation into myofibroblasts. These findings could lead to new strategies for lung cancer management.

**Supplementary Information:**

The online version contains supplementary material available at 10.1186/s13046-021-02206-z.

## Background

The relationship between embryonic and tumor cells has long been of interest to investigators. The earliest study, by Durante and Cohnheim in the nineteenth century [1, 2], proposed the Embryonic Rest Hypothesis, which suggested that the remains of embryonic cells in adult tissues could cause the formation of malignant tumors. This theory gave rise to subsequent studies exploring whether the embryonic environment could control tumor growth, reprogramming and differentiation. In the first known experimental study, the Rous sarcoma virus was injected into chicken embryos and into adult chickens. The embryos did not develop sarcoma, while the adults did [3–5]. Later studies in chimeric mice found that injecting embryonic carcinoma cells into the blastocyst [6, 7] or the uterus of pregnant mice [8, 9] caused the cells to lose their malignant capacity – known as “stemness” – and become differentiated tissue. More recently, similar results were found when human malignant melanoma cells were injected into the neural crest of chickens [10] or transplanted into zebrafish embryos [11, 12]. The four master embryonic transcription factors are octamer-binding transcription factor-3/4 (OCT-3/4), SRY-box transcription factor 2 (SOX2), Kruppel-like factor 4 (KLF4) and c-MYC. In the early phases of embryonic development, these master transcription factors, known collectively as OSKM, play a fundamental role in the regulation of embryonic cell self-renewal and in the capacity to turn back the biological clock and reprogram somatic cells to pluripotency [13, 14]. In vitro studies have shown that melanoma and breast cancer cells lose their aggressive capacity when cultured in human embryonic stem cell (hESC)-conditioned medium [10–15]. Taken together, these findings indicate that the embryonic environment is capable of reprogramming the phenotype of both somatic and tumor cells and that our DNA is not hermetic, immovable and unidirectional but rather has a high degree of plasticity, making it receptive to the stimuli it receives.

Cellular crosstalk between tissues is essential to the development of the embryonic lung. During the pseudoglandular stage of development (at weeks E9, E10 and E11), crosstalk between the mesodermal cells surrounding the endoderm promotes bronchial branching and the organization of the lung mesenchyme [16, 17]. The OSKM transcription factors play a crucial role in this process: OCT-3/4 acts to maintain the totipotency of the endodermal cells [18]; SOX2 and NK2 homeobox 1 (NKX2-1; also known as thyroid transcription factor 1 [TTF-1]), promote the differentiation of the proximal and ventral endoderm [19, 20]; and KLF4 promotes mesodermal differentiation [21, 22]. In contrast, the abnormal expression of these same transcription factors in the adult lung is related to tumorigenesis. Overexpression of OCT-3/4, SOX2 and c-MYC in patients with non-small-cell lung cancer (NSCLC) is associated with a decrease in cellular differentiation, an increase in metastases and poor prognosis [23–25]. NKX2-1 and KLF4 can act as oncogenes [26, 27] or tumor suppressors [28, 29] depending on the type of tissue and the microenvironment.

Based on the fact that the embryonic development of the lung generates the correct signals for the formation and correct differentiation of the adult lung, we hypothesized that the malignant phenotype of lung cancer cells could acquire benign characteristics when in contact with an embryonic lung microenvironment. To explore this hypothesis, we analyzed two NSCLC cell lines: A549 and H1299. These cell lines express OSKM, the hESC markers of pluripotency [30, 31], as well as high levels of NKX2-1 and lung surfactant proteins (SFTPs) [31, 32]. The cell lines were cultured in pseudoglandular lung mesenchyme-conditioned medium and the expression levels of OSKM, NKX2-1 and lung SFTPs were analyzed. Changes in expression level were correlated with changes in cell morphology, growth and differentiation.

## Methods

### Isolation and characterization of human embryonic mesenchymal lung (hEML) cells

Human embryonic lungs were obtained from terminations of pregnancy donated to the Body Donation Service of the Department of Human Anatomy and Embryology of the School of Medicine of the University of Barcelona for morphological and molecular studies. Lungs were dissected from embryos in the pseudoglandular stage of development (weeks E9, E10 and E11). The lungs were treated with dispase II (Sigma-Aldrich, St. Louis, MO, USA) and the lung mesenchyme was separated from the bronchial tree. Cell images were taken by IX53 inverted microscope using cellSense Entry 1.7 software (Olympus, Center Valley, PA, USA). Mesenchymal cells were fixed in 4% paraformaldehyde (Electron Microscopy Sciences, Hatfield, PA, USA) in Dulbecco’s Phosphate Buffered Saline (DPBS 1x) (Invitrogen, Carlsbad, CA, USA) and immunostained using monoclonal mouse anti-vimentin clone V9 ready-to-use and monoclonal mouse anti-human e-cadherin clone NCH-38 ready-to-use (Dako Denmark A/S, Glostrup, Denmark) in order to confirm the mesenchymal characteristics of the cells.

### Preparation of the human embryonic mesenchymal lung-conditioned medium (hEML-CM) and tumor-conditioned medium (T-CM)

**Fig. 1:**
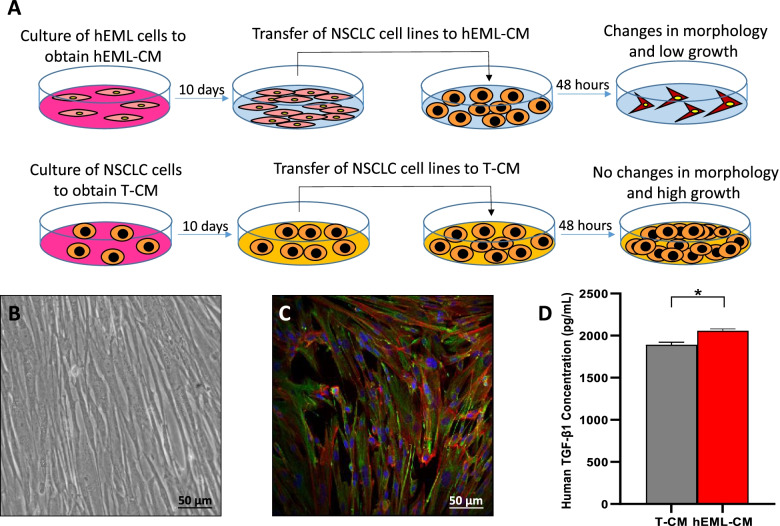
Conditioned media. **a** Preparation of the human embryonic mesenchymal lung conditioned medium (hEML-CM) and the control tumor-conditioned medium (T-CM). **b** Phase-contrast images of culture containing mesenchymal lung cells at 9 weeks (E9) of embryonic development. **c** Positive vimentin staining of E9 mesenchymal lung cells. **d** TGF-β1 levels in hEML-CM and T-CM as measured in the cell culture supernatants by ELISA. Data represent the mean ± SEM from at least three separate experiments. *p < 0.05

Figure 1a depicts the methodology used in the preparation of the CM. The hEML cells were cultured for 10 days in Dulbecco’s Modified Eagle Medium (DMEM) supplemented with 10% Fetal Bovine Serum (FBS) (Invitrogen) and 1% Penicillin/Streptomycin (PS) (Sigma-Aldrich), and grown under the recommended conditions of 37 °C in 5% CO_2_ and 95% relative humidity. The resulting hEML-CM was used in further analyses.

The human lung adenocarcinoma cell lines A549 and H1299 (American Type Culture Collection, Manassas, VA, USA) were cultured for 10 days: A549 in DMEM and H1299 in Roswell Park Memorial Institute 1640 medium (RPMI 1640) (Invitrogen), both supplemented with 10% FBS and 1% PS and grown at 37 °C in 5% CO_2_ and 95% relative humidity. The resulting T-CM was used as control medium in further analyses.

Exosome depletion was performed on day 10 in both hEML-CM and T-CM, as previously described [33, 34], by sequential centrifugation at 4 °C (300G 5′, 2500G 20′, 10,000G 30′) followed by ultracentrifugation 100,000G 2h. The hEML-CM and T-CM, with and without exosomes, were used in further analyses.

### Analysis of hEML-CM and T-CM

The hEML-CM and T-CM were analyzed with Human TGF-β1 Quantikine ELISA Kit (R&D Systems, Minneapolis, MN, USA) according to the manufacturer’s protocol for the analysis of TGF-β1 levels. Briefly, all reagents, standard dilutions and activated samples were prepared and 50 μL of assay diluent RD1-21 and 50 μL of control and activated samples were added to each well and incubated 2 h at room temperature (RT). Samples were then removed and washed three times. Next, 100 μL of TGF-β1 conjugate was added to each well and incubated 2 h at RT, after which the reagent was removed and washed three times again. Then 100 μL of substrate solution was added to each well and incubated 30 min at RT protected from light; 100 μL of stop solution was added to each well. Finally, after 30 min, the optical density was determined using VersaMax Tunable Microplate Reader (Molecular Devices, San Jose, CA, USA) to 450 nm.

### Transmission electron microscopy

Cells were fixed in 4% paraformaldehyde in DPBS 1x, post-fixed in 1% osmium tetroxide and 0.8% potassium ferrocyanide, dehydrated with acetone, and flat-embedded in epoxy resin. Ultrathin sections for transmission electron microscopy were cut and stained with 2% uranyl acetate for 10 min and with a lead-staining solution for 2 min. Images from stained ultrathin sections were acquired using a JEOL JEM-1010 transmission electron microscope coupled with a Gatan Orius SC1000 (model 832) digital camera.

### Neutralization of TGF-β1

TGF-β1 levels in the medium were neutralized using 3.75 μg/mL of TGF beta-1,2,3 Monoclonal Antibody (1D11) (Invitrogen). 5 × 10^4^ cells were cultured in a 12-well plate with or without the neutralizing antibody and the morphological study was performed at 48 h.

### Scratch wound healing assay

Proliferation was evaluated by a scratch wound healing assay using culture-insert 2 well in μ-Dish 35 mm (Ibidi, Gräfelfing, Germany) according to the manufacturer’s protocol. Briefly, 70 μL of 3 × 10^5^ cells/mL cell suspensions was applied in each well and incubated 24 h under the recommended conditions. Differences between cells growing in hEML-CM and T-CM were assessed with images taken at 6, 24 and 48 h using IX53 inverted microscope and analyzed using cellSense Entry 1.7 software.

### Clonogenic assays (2D and 3D)

In colony formation assay (2D) one hundred cells were cultured for each condition in 6-well plates during 12 and 15 days for A549 and H1299, respectively. At the end of the experiment colonies were stained with 0.5% crystal violet (Sigma-Aldrich) and cell colonies were counted under the stereoscopic microscope.

For Soft-agar assay (3D) Cell Transformation Assay Kit (Colorimetric) (ab235698, Abcam, Cambridge, MA, USA) was used following manufacturing recommendations. Briefly, 2 × 10^4^ cells were cultured in soft-agar during 8 days. At the end of the experiment cell number was quantified by spectrophotometry at 450 nm after addition of the WST Working Solution provided. Representative images were obtained under the microscope after addition of the Staining Solution provided in the kit.

### In vivo xenograft study

A xenograft tumor model was generated to evaluate differences in tumor formation and growth of control and conditioned A549 cells. 5-week-old male athymic nude mice (Rj:ATHYM-Foxn1nu/nu) were purchased (Janvier), and housed in sterile cages under laminar airflow hoods in a local SPF experimental animal facility with a 12 h light/dark cycle and constant temperature (to about 25 °C) and relative humidity (to about 50%). All animals were allowed to have free access to normal mouse chow and water.

A549 T-CM cells resuspended in PBS and A549 hEML-TM cells resuspended in hEML-CM were subcutaneously inoculated (1 × 10^6^ cells/100 μl) into both flanks. Three biological replicates (with 2 technical replicates per sample) were used for each genotype. Tumor volume was measured by a caliper every 4 days, and their volumes were calculated using the following equation: tumor volume (mm^3^) = 0.5 × length × width^2^. Mice were anesthetized and intracardially perfused with 4%PFA 35 days after inoculation. Tumors were dissected and measured at the end of the study.

### RNA extraction and gene expression analysis

Total RNA was isolated from cell lines using TRIzol Reagent (Life Technologies, Grand Island, NY, USA) according to the manufacturer’s protocol. The High Capacity cDNA Reverse Transcription Kit (Applied Biosystems, Foster City, CA, USA) was used to obtain cDNA using 500 ng of total RNA. Relative expression was determined by real-time polymerase chain reaction (qRT-PCR) using the StepOne Real-Time PCR System (Applied Biosystems). Taqman Gene Expression Assays (Life Technologies) were used to quantify the expression of OCT-3/4 (Hs04260367_gH), SOX2 (Hs01053049_s1), KLF4 (Hs00358836_m1), c-MYC (Hs00153408_m1), NKX2-1 (Hs00968940_m1), SFTPA (Hs01652580_g1), SFTPB (Hs00167036_m1), SFTPC (Hs00951326_g1) and SFTPD (Hs01108490_m1), DNMT3A (Hs01027166_m1), DNMT3B (Hs00171876_m1), MYH1 (Hs00428500_m1), MYH2 (Hs00430042_m1), MYH4 (Hs00757977_m1), MYH7 (Hs00165276_m1), MYH16 (Hs01385213_m1). Relative quantification was calculated using 2^-ΔΔCt^. CDKN1β (Hs01597588_m1) was used as endogenous control.

### Oil red O solution staining

In order to assess the presence or absence of lung SFTPs, the A549 and H1299 cells were stained with Oil Red O Solution (Sigma-Aldrich). On day 0, 5 × 10^4^ cells were seeded in 12-well plates. Cells were fixed with 4% paraformaldehyde in DPBS 1x and stained with Oil Red O Solution after 48 h of culture in hEML-CM or T-CM. Cell images were taken by IX53 Inverted Microscope using cellSense Entry 1.7 software.

### Immunofluorescence

Cells seeded on coverslips were fixed in 4% paraformaldehyde in DPBS at RT followed by permeabilization with 0.5% of Triton X-100 (Santa Cruz Biotechnology, Dallas, TX, USA) in DPBS 1x (PBTx) at RT for 5 min. Fixed cells were then blocked with 1% Bovine Serum Albumin (BSA) (Sigma-Aldrich) and 5% Normal Donkey Serum (NDS) (Sigma-Aldrich) in PBTx for 30 min at RT in darkness and incubated with primary antibodies diluted in blocking solution overnight at 4 °C in darkness. After primary antibody washing, secondary antibodies diluted in blocking solution were incubated for an hour at RT in darkness. Cells were stained and mounted on microscope slides using Fluoroshield with DAPI (Sigma-Aldrich). Immunostaining was performed using the following primary antibodies: rabbit anti-vimentin (1:500; Proteintech, Manchester, UK), mouse anti-alpha smooth muscle actin (α-SMA) (1:1000; Abcam) and mouse anti-myosin 4 (1:500; Invitrogen). Secondary antibodies were conjugated to Alexa Fluor 488 or Alexa Fluor 594 (ThermoFisher Scientific, Waltham, MA, USA). Immunofluorescence analysis was performed using Olympus BX51 Fluorescence Microscope (Olympus) and cell images were acquired by DPController and DPManager software (Olympus).

### Statistical analyses

Paired t-tests were used for comparisons between cells cultured in hEML-CM and those cultured in T-CM. ANOVA test for repetitive measures was used for comparisons between A549 T-CM and A549 hEML-TM tumor xenografts. All statistical analyses were performed using GraphPad Prism 8.

### Transcriptomic analysis

Affymetrix Clariom S Human arrays have been performed in the IDIBAPS Functional genomics core facility using three samples per group of each conditioned experiment. Raw .cel files were provided which were used for further analysis. Moreover, public available data from GEO database (https://www.ncbi.nlm.nih.gov/geo/) and ArrayExpress (https://www.ebi.ac.uk/arrayexpress/) were used for the following samples: HBEC6-KT (a normal immortalized cell line derived from lung bronquial epithelial cells) (GSE150541); primary human pulmonary artery endothelial cells (HPAECs) (GSE125508); Myoblast (GSE121023); and human lung fibroblasts (HLF) (E-MTAB-8488); A549 (GSE181088) and H1299 (GSE99993). Raw .cel files were downloaded for further analysis.

Bioiformatic analysis was peformed using R version 4.1.1 and Bioconductor 4.1.0. Package *oligo* was used for raw data processing and normalization with *rma* method. *removeBatchEffect* function from *limma* package was used to remove batch effects. Multidimensional scaling plot was performed using *plotMDS* function from *limma*. Distance matrix computation was done using *dist* function from *stats* package and graphically represented using *pheatmap* package. Differential expression analysis was performed using *limma* package and considering all genes with an adjusted *p*-value < 0.001. Venn diagrams were ploted using *VennDiagram* package. Hierarchical cluster analysis (Euclidean distance) of top 100 differentially expressed genes (DEG) were ploted using *pheatmap* package. Enrichment analysis for Gene Ontology (GO) terms was performed using *topGO* package from Bioconductor.

## Results

### E-cadherin and vimentin in embryonic cells and in hEML-CM

We analyzed the presence of E-cadherin and vimentin in human embryos in the pseudoglandular stage of development prior to obtaining embryonic mesenchymal cells. Confocal microscopy revealed that the mesodermal cells surrounding the endodermal cells were vimentin-positive while the endodermal cells themselves were E-cadherin-positive (Fig. S1). Once the mesenchymal cells had been separated from the epithelial cells, we analyzed the expression of vimentin in the mesenchymal cells and the presence of TGF-β1 in the culture. The mesenchymal cells displayed the morphological characteristics of mesodermal cells (Fig. 1b), with a high expression of vimentin (Fig. 1c). In addition, TGF-β1 expression was higher in the hEML-CM than in the T-CM (*p* = 0.013) (Fig. 1d).

### Morphological changes in NSCLC cells cultured in hEML-CM

**Fig. 2:**
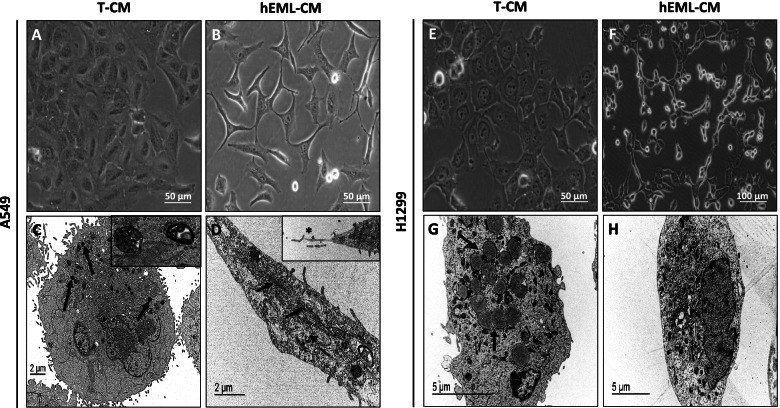
Comparison of morphological changes in A549 and H1299 cells cultured in hEML-CM and T-CM. **a** Morphology of A549 cultured in T-CM. **b** Morphology of A549 cultured in hEML-CM. Images were obtained with a phase-contrast microscope. **c** Ultrastructure of A549 cultured in T-CM, the cytoplasm contains densely stained lamellar bodies (arrows) inset shows detail of lamellar bodies. **d** Ultrastructure of A549 cultured in hEML-CM, shows spindled cell with cytoplasmatic filaments (arrows) and dilated rough endoplasmic reticulum (dotted arrow) inset shows detail of like fibronexus structure (asterisk). **e** Morphology of H1299 cultured in T-CM. **f** Morphology of H1299 cultured in hEML-CM. Images were obtained with a phase-contrast microscope. **g** Ultrastructure of H1299 cultured in T-CM, showing cytoplasmatic electron-dense lipid structures (arrows). **h** Ultrastructure of H1299 cultured in hEML-CM

Morphological changes were observed in the A549 and H1299 cells after 24 h in the hEML-CM but not in the T-CM (Fig. 2). No significant changes in results were observed between the hEML-CM with and without exosomes or between the T-CM with and without exosomes, indicating that the differences in results between the hEML-CM and the T-CM were not due to the presence or absence of exosomes.

While the A549 cells cultured in T-CM showed no morphological changes (Fig. 2a), those cultured in hEML-CM lost their intercellular junctions and acquired spindle-cell or stellate-cell morphology (Fig. 2b). The A549 cells cultured in T-CM were round and displayed multilamellar bodies (MLBs) in their cytoplasm (Fig. 2c), while those cultured in hEML-CM did not have MLBs and displayed microfilaments similar to track-like fibronexus structures (Fig. 2d).

The H1299 cells cultured in T-CM grew in a single layer and displayed no morphological changes (Fig. 2e), while those cultured in hEML-CM lost their round morphology and formed long interconnected extensions (Fig. 2f). Those cultured in T-CM showed abundant lipid structures (Fig. 2g), which were not observed in those cultured in hEML-CM (Fig. 2h).

Finally, we performed a TGF-β1 neutralizing experiment and evaluated the morphology changes in both cell lines (Fig. S2). After neutralization of TGF-β1 in the hEML-CM no relevant morphological changes were observed in any of the cell lines.

### Inhibited cell proliferation and migration in NSCLC cells cultured in hEML-CM

**Fig. 3:**
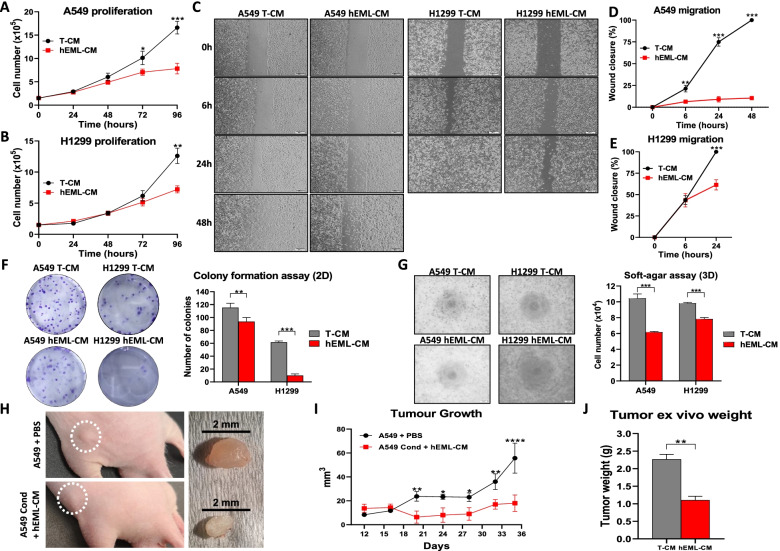
Functional in vitro and in vivo analysis of lung cancer cells cultured in T-CM and in hEML-CM. **a, b** Proliferation of A549 and H1299 cells after 96 h in T-CM and hEML-CM. Error bars represent the standard deviation of three replicates. **c** Wound healing assay in the A549 and H1299 cell lines. Cells were grown to confluence, wounded and grown in T-CM and hEML-CM, and photographed at 0, 6, 24 and 48 h. **d, e** Calculated wound healing areas in A549 and H1299 cells cultured in T-CM and hEML-CM. Data represent the mean ± SEM from at least three separate experiments at 0 h, 6 h, 24 h and 48 h. **f** Colony formation assay (2D) in the A549 and H1299 cell line. A representative image is shown in the left and the quantification of the colony number for each condition is shown in the right. Data represent the mean ± SEM from six separate experiments. **g** Soft-agar assay (3D) in the A549 and H1299 cell lines. A representative image is shown in the left and the quantification of cell number for each condition is shown in the right. **h** Representative image of 5-week-old male athymic nude mice (Rj:ATHYM-Foxn1nu/nu) with control or conditioned A549 cells injected in the flank at day 35 and resected tumors from each animal. **i** Graph showing the mean tumor volume of 3 biological replicates measured by a caliper every 4 days in both conditions. **j** Mean tumor weights in each condition at day 35. All data represent the mean ± SEM. *p < 0.05; **p < 0.01; ***p < 0.001; ****p < 0.0001

At 48 h, the A549 cells cultured in hEML-CM showed a slightly decreased proliferation compared to those cultured in T-CM. This difference in cell proliferation was significant at 72 h (*p* = 0.04) and 96 h (*p* < 0.001) (Fig. 3a). At 48 h, the H1299 cells cultured in hEML-CM showed no difference in proliferation compared to those cultured in T-CM. However, at 72 h, there was a slight difference in proliferation, which was significant at 96 h (*p* = 0.001) (Fig. 3b).

The scratch wound healing assay showed that the A549 cells cultured in hEML-CM maintained their mesenchymal morphology and were not confluent at 48 h (Fig. 3c and d). Mesenchymal cells migrate but proliferate very slowly, and these A549 cells migrated towards the center of the scratch wound as in the healing process (Fig. S3). In contrast, the A549 cells cultured in T-CM were completely confluent at 48 h (Fig. 3c). At 24 h, the H1299 cells cultured in hEML-CM were not confluent but those cultured in T-CM were (*p* < 0.001). At 48 h, the H1299 cells were confluent in both in hEML-CM and T-CM, indicating that the H1299 cell line has a higher cell proliferation rate than the A549 cell line (Fig. 3c and e).

### Inhibited clonogenic capacity in NSCLC cells cultured in hEML-CM

Clonogenic capacity in 2D and 3D was evaluated using colony formation assay and soft agar assay respectively. Colony formation assay at 12 days, the A549 cells cultured in hEML-CM showed a mean decrease of 18.5% in the number of colonies compared to those cultured in T-CM (*p* = 0.002; Fig. 3f). At 15 days, the H1299 cells cultured in hEML-CM showed a mean decrease of 83.7% in the number of colonies compared to those cultured in T-CM (*p* < 0.001; Fig. 3f).

In Soft agar-assay at 8 days, the cells cultured in hEML-CM showed a mean decrease of 40.9% for A549 (*p* < 0.001) and 20% for H1299 (*p* < 0.001) in the number of cells compared to those cultured in T-CM (Fig. 3g).

### Inhibited in vivo tumor growth of NSCLC cells cultured in hEML-CM

To evaluate the capacity of conditioned cell to growth in vivo, we injected control and conditioned A549 cells subcutaneously in the flank of athymic nude mice and measure the volume each 4 days until day 35. We observed that conditioned medium exerted a significant inhibitory effect on tumor volume in vivo compared with the control group that can be observed from day 16 to 35 (*p* < 0.05 for all measured points; Fig. 3i). Tumors were resected at day 35 from tumor-bearing mice, and a representative image of the flank and of the resected tumor in control and conditioned group can be observed in Fig. 3h. The weight measure of resected tumors revealed to be significantly lower in the hEML-CM treated group compared with the control group (*p* = 0.0033; Fig. 3j).

### Downregulation of OSKM, NKX2-1 and SFTPs, and upregulation of DNMT3A/3B in NSCLC cells cultured in hEML-CM

**Fig. 4:**
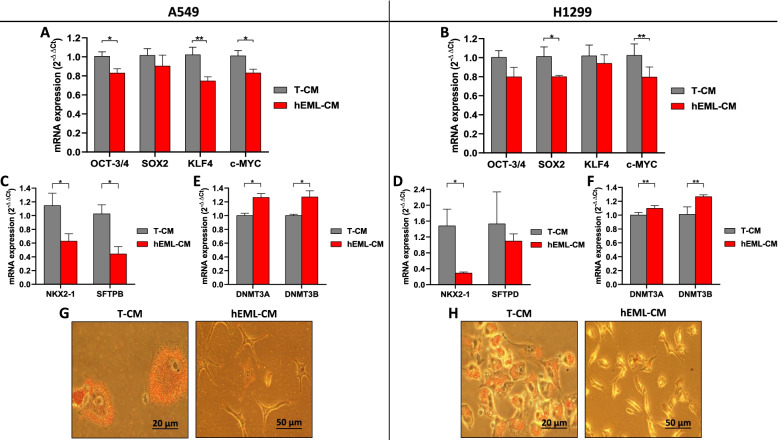
Expression of OSKM, NKX2-1, SFTPs and DNMT3A/B in A549 and H1299 cells cultured in T-CM and hEML-CM. **a** mRNA expression levels of OSKM transcription factors in A549 cells cultured in T-CM or hEML-CM. Differences were observed between the cultures for OCT-3/4, KLF4 and c-MYC but not for SOX2. **b** mRNA expression levels of OSKM transcription factors in H1299 cells cultured in T-CM or hEML-CM. Differences were observed between the cultures for SOX2 and c-MYC. **c** A549, mRNA expression levels of NKX2-1 and SFTPB. **d** H1299; mRNA expression levels of NKX2-1 and SFTPD. **e** A549, mRNA expression levels of DNMT3A and DNMT3B. **f** H1299, mRNA expression levels of DNMT3A and DNMT3B. Data represent the mean ± SEM from three separate experiments. **g, h** Phase contrast images of Oil Red O Solution staining of lipid droplets in A549 and H1299 cells lines, respectively, showing positive staining in T-CM and negative staining in hEML-CM. *p < 0.05; **p < 0.01

In the A549 cells cultured in hEML-CM, we observed a significant downregulation of OCT-3/4 (*p* = 0.02), KLF4 (*p* = 0.003) and c-MYC (*p* = 0.014), compared to the cells cultured in T-CM, while the downregulation of SOX2 was not significant (Fig. 4a). In the H1299 cells cultured in hEML-CM, only SOX2 (*p* = 0.045) and c-MYC (*p* = 0.009) were significantly downregulated compared to the cells cultured in T-CM, while the downregulation of OCT-3/4 and KLF4 was not significant (Fig. 4b).

In order to explore the potential relationship between NKX2-1 levels and the synthesis of lung SFTPs, we analyzed the mRNA expression levels of NKX2-1, SFTPA, SFTPB, SFTPC and SFTPD. NKX2-1 was downregulated in both A549 (*p* = 0.029) and H1299 (*p* = 0.041) cells cultured in hEML-CM but upregulated in those cultured in T-CM (Fig. 4c and d). SFTPB was downregulated in A549 (*p* = 0.022) cells cultured in hEML-CM and upregulated in those cultured in T-CM (Fig. 4c). SFTPD was downregulated in H1299 cells cultured in hEML-CM compared to those cultured in T-CM but the difference was not significant (Fig. 4d). SFTPA and SFTPC were not expressed in either of the cell lines.

We then analyzed the expression levels of DNA methyltransferase 3A (DNMT3A) and DNA methyltransferase 3B (DNMT3B) to explore changes in genes controlling methylation. Both DNMT3A and DNMT3B were upregulated in the A549 cells (*p* = 0.036 and *p* = 0.042, respectively) and in the H1299 cells (*p* = 0.003 and *p* = 0.007, respectively) cultured in hEML-CM compared to those cultured in T-CM (Fig. 4e and f).

In order to detect the surfactant lipid, the A549 and H1299 cells were stained with Oil Red O Solution. Both cell lines cultured in T-CM had a high degree of cytoplasmic staining, while those cultured in hEML-CM showed no staining (Fig. 4g and h).

### Expression of myofibroblast markers and myosin 4 (MYH4) in NSCLC cells cultured in hEML-CM

**Fig. 5:**
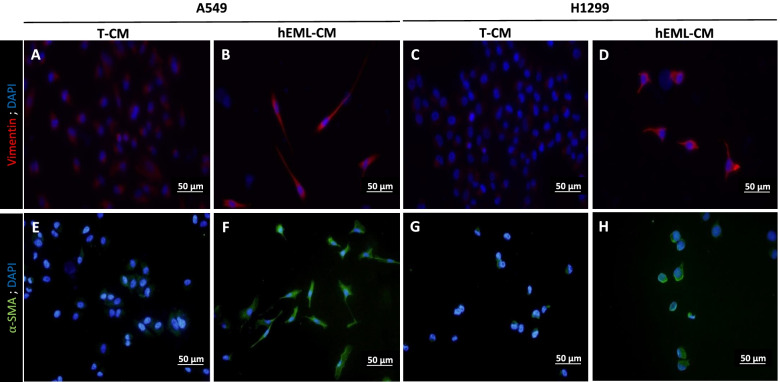
Immunofluorescence detection of vimentin and α-SMA in A549 and H1299 cells cultured in T-CM and hEML-CM. **a-d** Vimentin in A549 and H1299 cells, **a, c** showing negative staining in cells cultured in T-CM and **b, d** showing positive staining in cells cultured in hEML-CM. **e-h** α-SMA in A549 and H1299 cells, **e, g** showing negative staining in cells cultured in T-CM and **f, h** showing positive staining in cells cultured in hEML-CM. Nuclei were counterstained with DAPI

The morphology of the A549 and H1299 cells cultured in hEML-CM was similar to that of myofibroblasts. In order to assess the presence or absence of myofibroblast markers in these cells, we analyzed vimentin and α-SMA. The A549 and H1299 cells cultured in hEML-CM showed a higher degree of staining of both markers than those cultured in T-CM (Fig. 5). Vimentin in A549 and H1299 cells, showing positive staining in cells cultured in hEML-CM (Fig. 5b and d), but not in cells cultured in T-CM (Fig. 5a and c). α-SMA in A549 and H1299 cells, showing positive staining in cells cultured in hEML-CM (Fig. 5f and h), but not in cells cultured in T-CM (Fig. 5e and g).

**Fig. 6:**
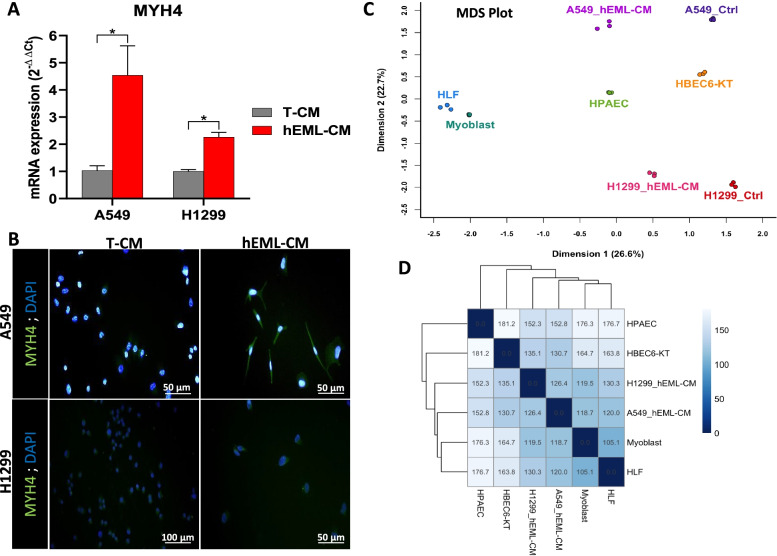
Evaluation of myofibroblastic differentiation of hEML-CM cells. **a** Upregulation of MYH4 mRNA in A549 and H1299 cells cultured in hEML-CM compared to those cultured in T-CM. Data represent the mean ± SEM from three separate experiments. **b** Immunofluorescence detection of MYH4 in A549 and H1299 cells cultured in T-CM and hEML-CM. In hEML-CM, the A549 cells showed highly positive staining, while the staining in H1299 cells was less intense. No staining was detected in cells cultured in T-CM. Nuclei were counterstained with DAPI. **c** Multidimensional Scaling plot of distances between gene expression profiles for A549_hEML-CM, A549_Ctrl, H1299_hEML-CM, H1299_Ctrl, HBEC6-KT, HPAEC, Myoblast and HLF samples. Three samples of each group have been included in the analysis. **d** Simplified distance matrix heat map showing the mean of the three replicates for each group. *p < 0.05

Since myofibroblasts also express myosin heavy chain (MHC) isoforms, we then analyzed the mRNA expression of MYH1, MYH2, MYH3, MYH4, MYH7 and MYH16 in these cells. MYH4 was upregulated in the A549 (*p* = 0.033) and the H1299 (*p* = 0.032) cells cultured in hEML-CM compared to those cultured in T-CM (Fig. 6a). MYH1, MYH2, MYH3, MYH7 and MYH16 were not expressed in either of the cell lines. The A549 cells cultured in hEML-CM showed highly positive staining for MYH4, while the staining in H1299 cells cultured in hEML-CM was less intense. Neither the A549 nor the H1299 cells cultured in T-CM showed staining for MYH4 (Fig. 6b).

### Transcriptomic analysis showed that hEML-CM cells approach to myoblasts and lung fibroblasts

In Fig. 6c are showed the results of the Multidimensional cluster analysis performed using the transcriptomic data. Not including hEML-CM cells, we can observe five basic groups from left to right in the graph (Dimension 1): mesenchymal group (HLF and myoblasts); endothelial group (HPAEC); normal epithelial group (HBEC6-KT); A549; and H1299. The closest group to both cancer cell lines was the normal epithelial group. However, the hEML-CM cells (A549 and H1299) moved away from their own control group and from normal epithelial cells, and approaches to the endothelial and mesenchymal groups. The distance matrix analysis revealed that the closest group to hEML-CM cell was the mesenchymal group (Fig. 6d and Fig. S4).

### Identification of DEGs between control and hEML-CM treated cells

**Fig. 7:**
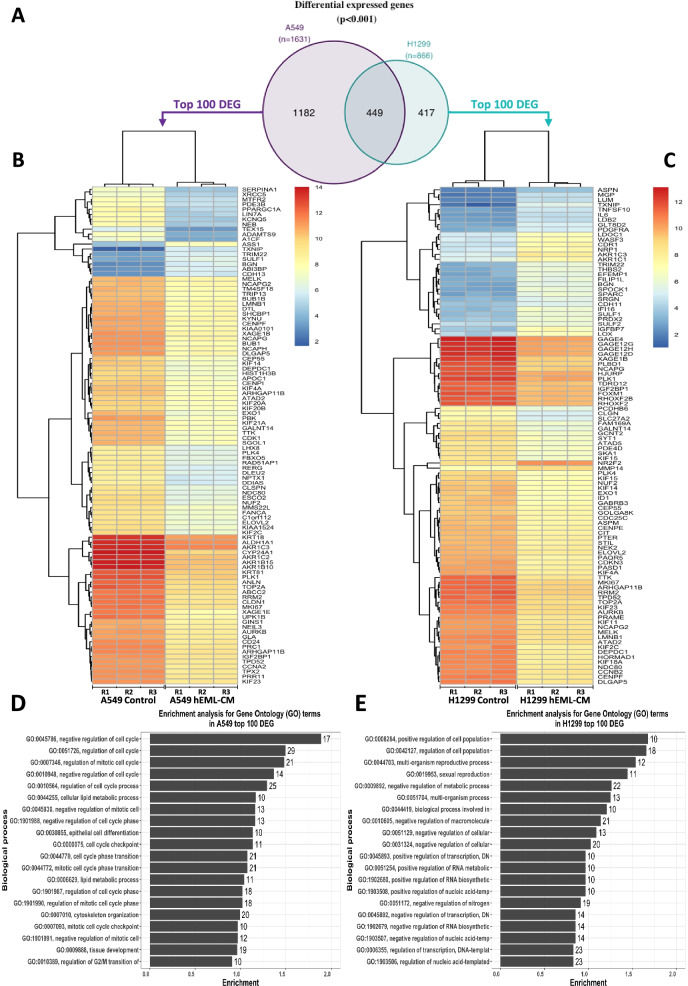
Transcriptomic analysis to evaluate the effect of hEML-CM. **a** Venn diagram showing the number of common differential expressed genes between control and hEML-CM treated cells in both cell lines. **b, c** Heat map of top 100 differentially expressed genes in A549 and H1299, respectively. **d, e** Enrichment analysis for Gene Ontology (GO) terms using the top 100 differentially expressed genes in A549 and H1299 cell lines. The numbers indicated in the bars indicate the number of genes included in each GO term

Differential expression analysis showed that 1631 and 866 genes were differentially expressed (*p* < 0.001; Table S1) in A549 and H1299 cells after treatment with hEML-CM, respectively (Fig. 7a). The top significant 100 DEG for each cell line are showed in Fig. 7b and c. The enrichment analysis for GO terms (Fig. 7d, e and Table S2) showed that the most significantly enriched GO terms for A549 were related to cell cycle regulation (GO terms: GO:0045786, GO:0051726) and for H1299 with proliferation regulation (GO:0008284, GO:0042127).

## Discussion

In his 1983 Rous-Whipple Award Lecture [35], the pathologist GB Pierce proposed for the first time that malignant tumors originated in the stem cells of healthy tissue. He further suggested that the same mechanisms that control tissue development during embryogenesis also controlled growth and differentiation of tumor stem cells. Following this line of research, in the present study, we have cultured two NSCLC cell lines (A549 and H1299) in hEML-CM and T-CM and explored whether hEML-CM could alter the malignant phenotype of the tumor cells. We observed that both the A549 and H1299 cells cultured in hEML-CM lost their malignant phenotype and acquired morphological and molecular characteristics similar to those of mesenchymal cells. Moreover, up to 1182 genes in A549 and 866 genes in H1299 were observed differentially expressed after treatment with hEML-CM. Previous studies had reported that cultures with or without fetal serum had a substantial differential impact on the phenotypes of A549 cells and of murine embryonic stem cells [36], where those cultured in a medium without fetal serum acquired characteristics of alveolar epithelial type II (ATII) cells, including the presence of MLBs in the cytoplasm [37–40]. In the present study, the A549 and H1299 cells cultured in hEML-CM lost their epithelial morphology, did not contain MLBs in the cytoplasm, and acquired characteristics of mesodermal cells, including the presence of vimentin and α-SMA, which are hallmarks of myofibroblasts [41, 42]. In contrast, the A549 and H1299 cells cultured in T-CM maintained their epithelial morphology. Moreover, since these morphological changes occurred regardless of whether the hEML-CM contained exosomes, we can speculate that soluble factors - not exosomes - are responsible for the changes.

In the pseudoglandular stage of development of the embryonic lung, the release of TGF-β1 by mesenchymal cells is essential to lung branching and alveolarization [43]. In our study, the hEML-CM contained high concentrations of TGF-β1, which could explain why the cells acquired the mesodermal characteristics and increased vimentin and α-SMA expression. In this line, our TGF- β1 neutralizing experiment (Fig. S2) showing that after inhibition of TGF-β1 in the hEML-CM no morphological changes were observed, reinforced the potential role of TGF-β1 in the acquisition of the mesodermal phenotype.

Previous studies showed that TGF-β1 induces the expression of both vimentin and α-SMA in myofibroblasts [41, 42]. During embryonic development, TGF-β1 expression is necessary for epithelial-mesenchymal transition (EMT), which is crucial to normal embryonic development [44]. In adult tissues, however, abnormal TGF-β1-induced EMT leads to tumor development and progression [43, 45]. In vitro studies have shown that when TGF-β1 is added to a culture medium, subpopulations of A549 cells begin EMT and cells with a malignant phenotype (stemness) undergo morphological changes, increase their growth, and overexpress pluripotent markers such as OCT-3/4 [38].

In our study, the A549 and H1299 cells cultured in hEML-CM decreased their growth and clonogenic capacity. In this line the results of the transcriptomic profile showed an enrichment of GO terms related with cell cycle and proliferation regulation. Moreover, the treated cells also underexpressed OCT-3/4, SOX2, KLF4 and c-MYC, leading to loss of pluripotency. These findings lead us to suggest that in addition to TGF-β1, other factors in hEML-CM must be involved in controlling the phenotype of the different clones present in the A549 and H1299 cell lines. In addition to the downregulation of the OSKM genes, we observed an upregulation of DNMT3A and DNMT3B in the cells cultured in hEML-CM, suggesting that the embryonic medium may contain an epigenetic control mechanism whereby DNMT3A/3B upregulation could silence the genes responsible for pluripotency and activate cell differentiation. This hypothesis is borne out by previous studies demonstrating that DNMT3A/3B cooperate in OCT-3/4 and NANOG promotor methylation during the differentiation of embryonic stem cells and carcinoma cells [46].

The transcription factor NKX2-1, which is essential for the correct development of the embryonic lung, is located in the terminal respiratory units of the epithelial cells [26], where it activates several genes involved in lung physiology, including the lung surfactants SFTPA, SFTPB, SFTPC and SFTPD [47]. NKX2-1 is expressed in 70% of lung adenocarcinomas and can act either as an oncogene or tumor suppressor [27, 48]. The A549 cells have characteristics similar to those of ATII cells, including the NKX2-1 mediated expression of lung surfactants. However, the presence of NKX2-1 in A549 cells is still not clear. Some studies have not detected NKX2-1 in A549 cells [49, 50], while others have detected the TTF-1 protein by immunohistochemistry [51] and high NKX2-1 mRNA expression by qRT-PCR [39, 52]. These contradictory findings may be due to the presence of various clones in the A549 cell line, each having different degrees of differentiation and different responses to external stimuli [38, 39]. Along these lines, we have observed a high level of NKX2-1 expression in the A549 and H1299 cells cultured in T-CM, while NKX2-1 expression was downregulated in the majority of cells cultured in hEML-CM. In addition, the assay with Oil Red O Solution showed lung SFTP staining in the cytoplasm of the A549 and H1299 cells that did not undergo morphological changes. In normal and tumor lung tissue, high NKX2-1 levels activate SFTP synthesis [47], but high TGF-β1 levels inhibit NKX2-1, leading to low SFTP expression [53]. In our study, low levels of NKX2-1 in the cells cultured in hEML-CM were associated with corresponding low staining of SFTPB in the A549 cells and of SFTPD in the H1299 cells. We can speculate that the presence of TGF-β1 in the hEML-CM is a determining factor in the inhibition of SFTPB and SFTPD and in the morphological transformation of the cells. Previous studies in this line have shown that TGF-β1 inhibits the synthesis of SFTPs through the interaction of SMAD3 with NKX2-1 [53, 54] and that TGF-β1 activates genes that are involved in the differentiation of lung fibroblasts into myofibroblasts [43, 55, 56].

In addition to vimentin and α-SMA, myofibroblasts express several MHC proteins [41, 57, 58]. For this reason, having observed that the A549 and H1299 cells cultured in hEML-CM had positive staining for vimentin and α-SMA, we then analyzed MHC isoform expression by immunofluorescence and qRT-PCR and found that the cells expressed only MYH4, which is expressed in humans after birth but not in embryos. However, we may well have detected MYH8 rather than MYH4, since 90% of the genetic sequence of MYH4 is the same as that of MYH8, which is only expressed in embryonic muscles [58, 59]. In line with these results, we observed that the transcriptomic profile of the hEML-CM cells approaches to myoblasts and to lung fibroblasts. All these results seem to indicate that the lung adenocarcinoma A549 and H1299 cells cultured in an embryonic lung medium begin to differentiate into myofibroblasts. The function of myofibroblasts depends on the microenvironmental stimuli they receive. In the adult lung, they are associated with several pathologies [60] and have been proposed as markers of good [61] or poor [62] prognosis in lung cancer. Along these lines, a recent study in human pancreatic ductal adenocarcinoma has shown that expression of α-SMA in myofibroblasts is associated with good prognosis, while low expression of α-SMA is linked to worse prognosis [63]. Additionally, in the pseudoglandular and canalicular stages of embryonic lung development, myofibroblasts expressing α-SMA [64, 65] and various MHC isoforms are essential for normal development of the alveolar system [66].

**Fig. 8:**
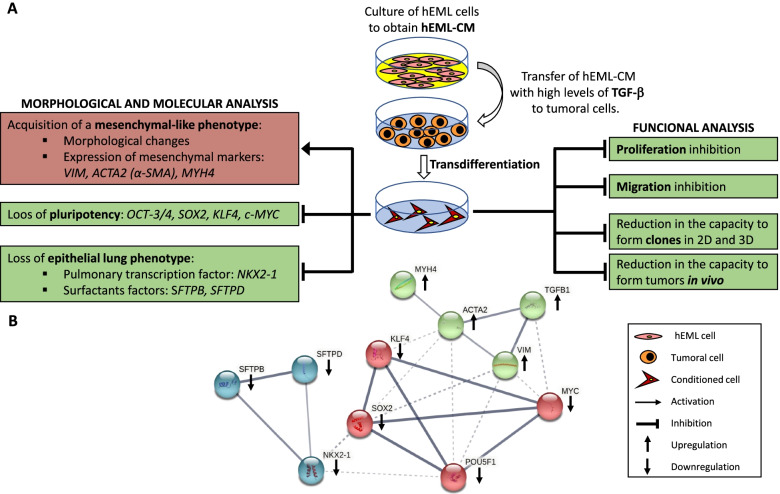
**a** Schema summarizing the main findings of the work and **b** String analysis (https://string-db.org) showing the relation between the analyzed genes. Color nodes are based on the results of the k-means clustering analysis. The arrows indicate the expression level in the hEML-CM cells

The current therapies in lung cancer and solid tumors in general are mostly focused in inducing apoptosis of highly proliferating tumor cells with the associated handicaps of acquisition of treatment resistance and normal cell-related toxicity. The present results could lead to think new strategies in terms of lung cancer treatment based on the idea of reducing self-replication of lung cancer cells by differentiating them using embryonic factors secreted during lung development. The paradigm of differentiation therapies is the use of retinoic acid in promyelocytic leukemia [67], which is an important gene during embryonic development. Although, differentiation therapies have been also attempted in other tumors with no relevant clinical results [68], here we are using a different analytical approach: the analysis of embryonic factors from the same organ to identify new molecules with potential treatment use. We have observed that transdifferentiating lung cancer cell lines using embryonic media from embryonic mesenchymal lung cells, significantly reduced their malignant capacities both in vitro and in vivo (summarized in Fig. 8). Our data serves as a foundation for future studies that will include the identification of the whole set of molecules responsible for the efficiency of this potential therapy and the evaluation of the potential clinical use.

## Conclusions

In summary, lung adenocarcinoma cells cultured in an embryonic lung medium lose their epithelial morphology, reduce the expression of the adenocarcinoma markers NKX2-1 and SFTPs, decrease proliferation, downregulate the expression of the OSKM transcription factors, upregulate the expression of DNMT3A/3B and develop mesodermal features that are characteristic of myofibroblasts. Taken together, these findings lead us to conclude that stimuli from the embryonic lung can modulate the malignant phenotype of lung cancer cells. Further studies are warranted to identify the specific embryonic stimuli influencing this process and to determine if there is an embryonic organotropic mechanism that controls tumorigenesis and growth. These findings could lead to new strategies for early detection and treatment of lung cancer tumors.

## Supplementary Information

**Additional file 1 : Figure S1.** Human embryonic lung. **a** Confocal image of human embryonic lung in the pseudoglandular stage (E9). **b** Detail showing intensive positive staining for E-cadherin in epithelial cells. **c** Detail showing positive staining for vimentin in mesenchymal cells. The mesenchymal cells were then isolated and cultured for the preparation of the hEML-CM.

**Additional file 2 **: **Figure S2.** Morphological study at 48 h after neutralizing TGF-β1 in A549 and H1299 cultured in T-CM and hEML-CM. When neutralizing antibody was used the morphological changes were considerably reduced in the hEML-CM condition.

**Additional file 3 **: **Figure S3.** Scratch wound healing assay in A549 cells cultured in hEML-CM. **a** At 6 h, the cells differentiate and migrate to the center of the scratch wound. **b** At 24 h and **c** 48 h, the cells continue to migrate but there is no cell growth to heal the wound.

**Additional file 4 **: **Figure S4.** Complete distance matrix heat map including all anlayzed samples.

**Additional file 5 **: **Table S1.** List of DEG between control and conditioned cells.

**Additional file 6 **: **Table S2.** GO terms identified in the enrichment analysis and the list of DEG genes included in each GO term.

## Data Availability

The datasets used and analyzed during the current study are available from the corresponding author on reasonable request.

## Abbreviations

NSCLC: Non-small-cell lung cancer
hEML-CM: Human embryonic mesenchymal lung-conditioned medium
OCT-3/4: Octamer-binding transcription factor-3/4
SOX2: SRY-box transcription factor 2
KLF4: Kruppel-like factor 4
OSKM: OCT-3/4, SOX2, KLF4 and c-MYC
hESC: Human embryonic stem cell
NKX2-1: NK2 homeobox 1
TTF-1: Thyroid transcription factor 1
SFTPs: Surfactant proteins
hEML: Human embryonic mesenchymal lung
DPBS 1x: Dulbecco’s phosphate buffered saline
T-CM: Tumor-conditioned medium
DMEM: Dulbecco’s modified eagle medium
FBS: Fetal bovine serum
PS: Penicillin/Streptomycin
RPMI 1640: Roswell Park Memorial Institute 1640 medium
qRT-PCR: Real-time polymerase chain reaction
PBTx: 0.5% of Triton X-100 in DPBS 1x
BSA: Bovine serum albumin
NDS: Normal donkey serum
α-SMA: Alpha smooth muscle actin
HPAEC: Primary human pulmonary artery endothelial cells
HLF: Human lung fibroblasts
DEG: Differentially expressed genes
GO: Gene Ontology
MLBs: Multilamellar bodies
DNMT3A: DNA methyltransferase 3A
DNMT3B: DNA methyltransferase 3B
MYH4: Myosin 4
MHC: Myosin heavy chain
ATII: Alveolar epithelial type II
EMT: Epithelial-mesenchymal transition
