# Supplementary figures and images for: Human embryonic mesenchymal lung-conditioned medium promotes differentiation to myofibroblast and loss of stemness phenotype in lung adenocarcinoma cell lines
Source: J Exp Clin Cancer Res. 2022 Jan 26;41:37. doi: 10.1186/s13046-021-02206-z (PMC8790861; doi:10.1186/s13046-021-02206-z)

## Slide 1
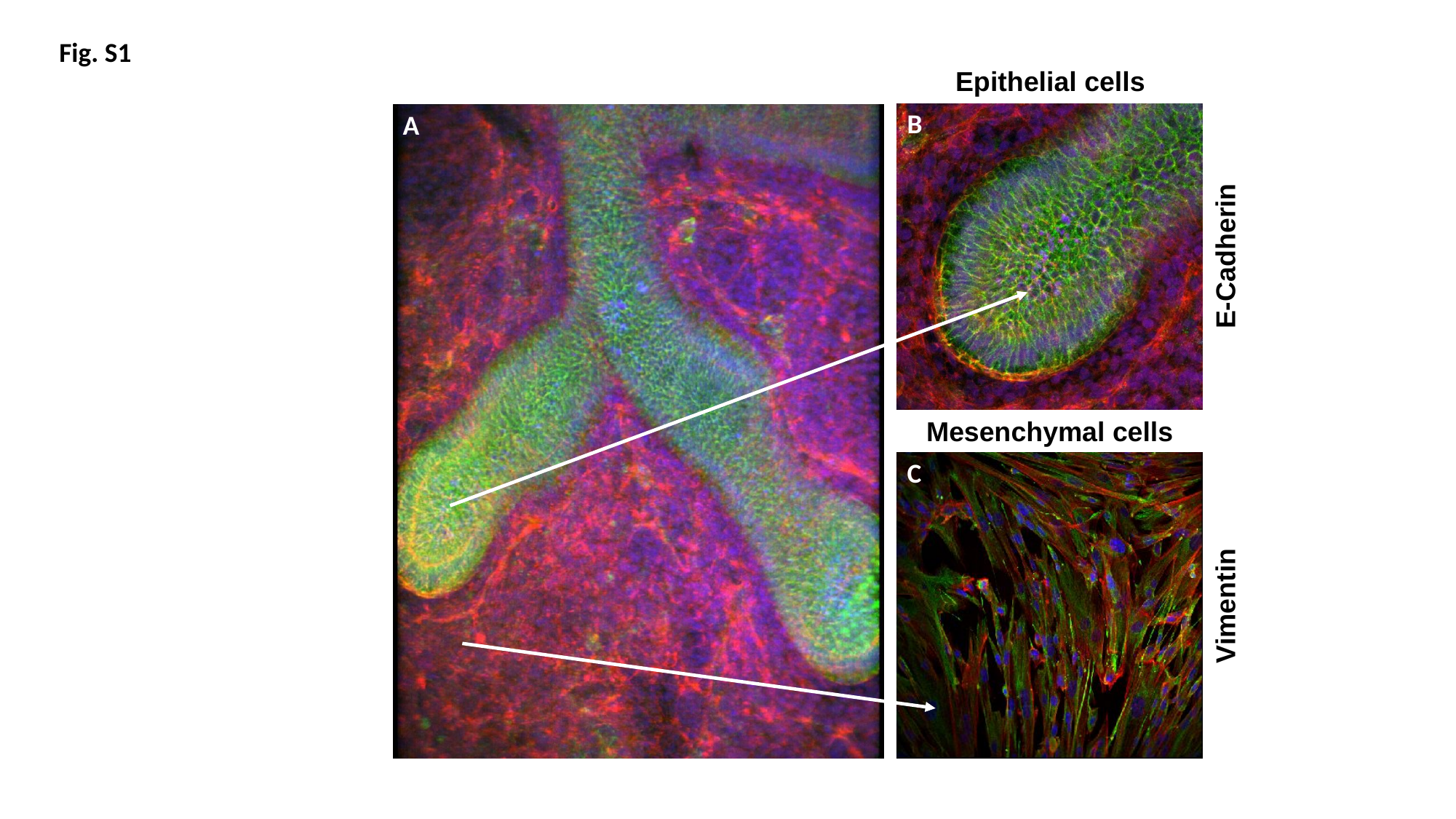

Fig. S1
Epithelial cells
B
A
C
E-Cadherin
Mesenchymal cells
Vimentin

Supplement: Supplementary file 1 — Additional file 1 : Figure S1. Human embryonic lung. a Confocal image of human embryonic lung in the pseudoglandular stage (E9). b Detail showing intensive positive staining for E-cadherin in epithelial cells. c Detail showing positive staining for vimentin in mesenchymal cells. The mesenchymal cells were then isolated and cultured for the preparation of the hEML-CM. [file 13046_2021_2206_MOESM1_ESM.pptx]

## Slide 1
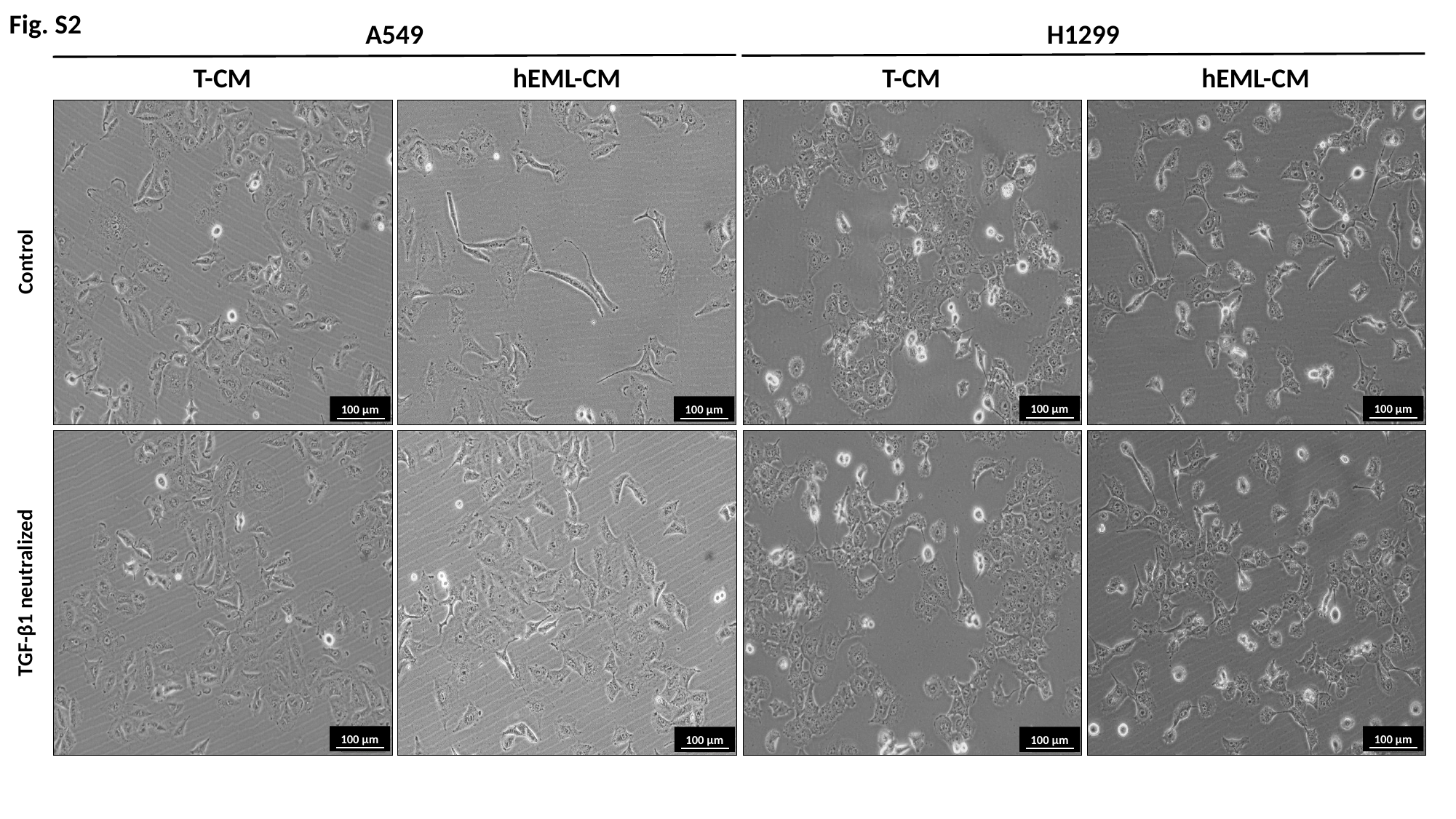

Fig. S2
A549
T-CM
hEML-CM
H1299
T-CM
hEML-CM
Control
TGF-β1 neutralized
100 µm
100 µm
100 µm
100 µm
100 µm
100 µm
100 µm
100 µm

Supplement: Supplementary file 2 — Additional file 2 : Figure S2. Morphological study at 48 h after neutralizing TGF-β1 in A549 and H1299 cultured in T-CM and hEML-CM. When neutralizing antibody was used the morphological changes were considerably reduced in the hEML-CM condition. [file 13046_2021_2206_MOESM2_ESM.pptx]

## Slide 1
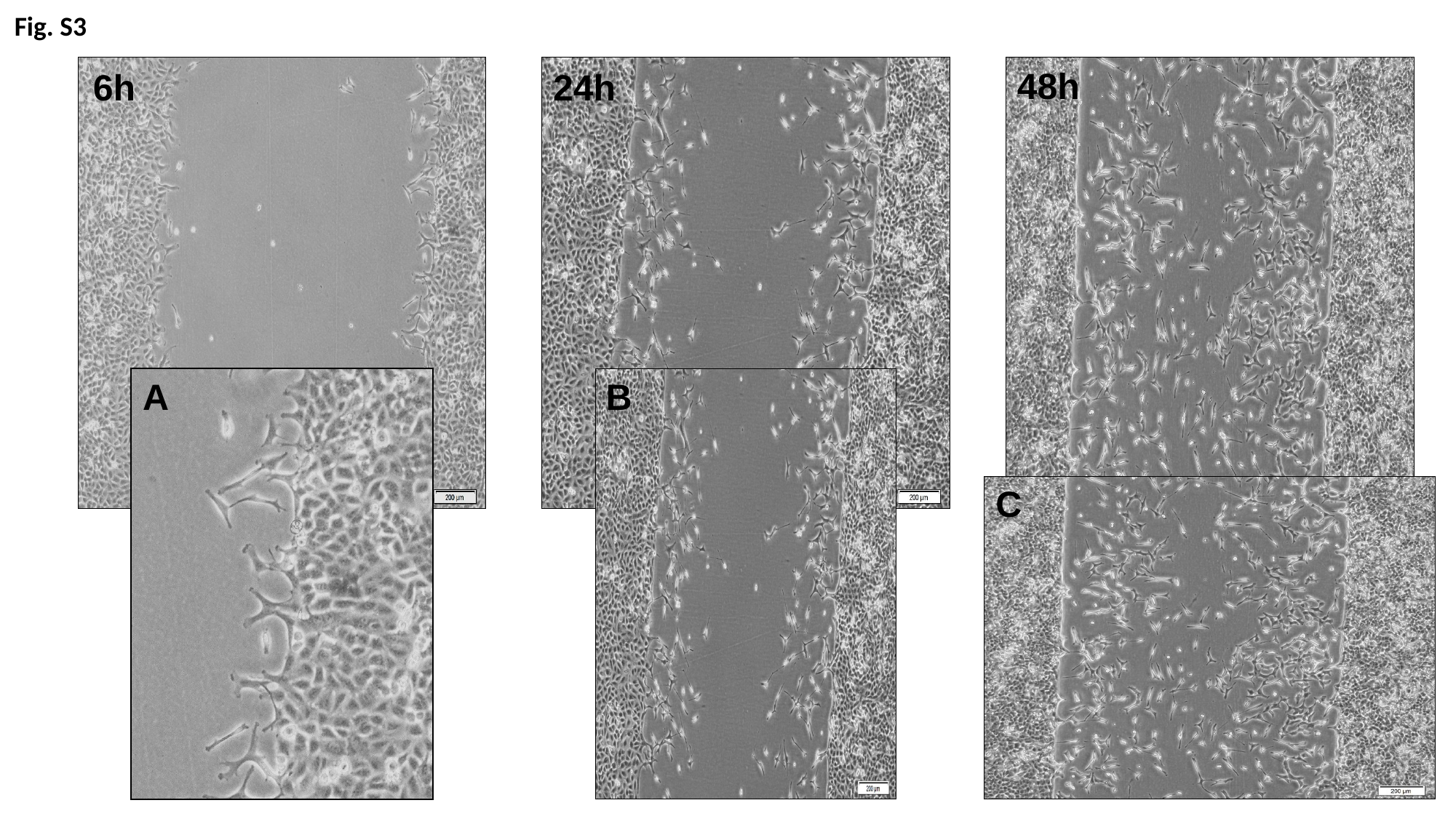

Fig. S3
48h
6h
24h
A
B
C

Supplement: Supplementary file 3 — Additional file 3 : Figure S3. Scratch wound healing assay in A549 cells cultured in hEML-CM. a At 6 h, the cells differentiate and migrate to the center of the scratch wound. b At 24 h and c 48 h, the cells continue to migrate but there is no cell growth to heal the wound. [file 13046_2021_2206_MOESM3_ESM.pptx]

## Slide 1
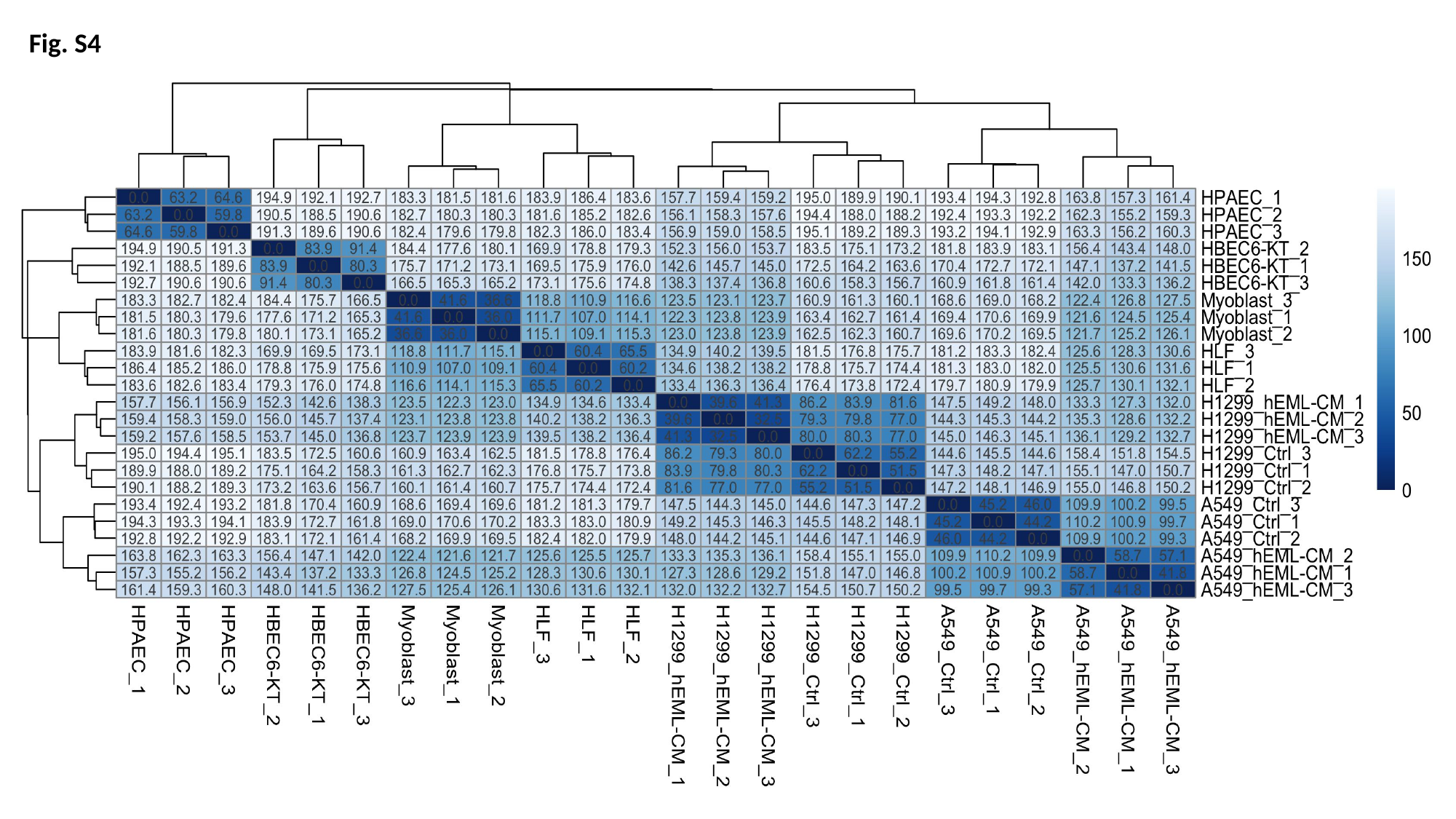

Fig. S4

Supplement: Supplementary file 4 — Additional file 4 : Figure S4. Complete distance matrix heat map including all anlayzed samples. [file 13046_2021_2206_MOESM4_ESM.pptx]
